# The influence of dirt track hardness on equine limb acceleration and impact attenuation

**DOI:** 10.1186/s12917-026-05376-0

**Published:** 2026-03-19

**Authors:** Olivia L. Bruce, Thilo Pfau, Laura E. Crack, Andrew Sawatsky, Renaud Leguillette, W. Brent Edwards

**Affiliations:** 1 Department of Biomedical Engineering, Schulich School of Engineering, University of Calgary, Calgary, AB Canada; 2 McCaig Institute for Bone and Joint Health, Cumming School of Medicine, University of Calgary, Calgary, AB Canada; 3 Faculty of Veterinary Medicine, University of Calgary, Calgary, AB Canada; 4 Human Performance Laboratory, Faculty of Kinesiology, University of Calgary, Calgary, AB Canada; 5 Department of Radiology, Stanford University, 1201 Welch Rd, Rm P093, Stanford, CA 94305 USA

**Keywords:** Track properties, Track hardness, Horse, Wearable sensors, Gallop

## Abstract

**Background:**

Track hardness is a modifiable factor that may influence musculoskeletal loading and injury risk in equine athletes. The purpose of this study was to evaluate the effects of track hardness on segmental accelerations and impact attenuation in the equine forelimb at racing speeds. We hypothesized that harder surfaces would result in greater peak segmental accelerations and reduced attenuation.

**Methods:**

Twelve Thoroughbred chuckwagon outriding horses, instrumented with tri-axial accelerometers and a global positioning system (GPS) unit, galloped on the Calgary Stampede dirt racetrack with different track preparations. Track hardness was varied through harrowing depth and moisture content and measured using a surface impact tester. Track conditions were grouped into soft (22.3–26.3 g), medium (38.1–44.3 g), and hard (61.2 g).

**Results:**

Peak resultant hoof impact accelerations were 19% greater on the medium tracks when compared to the softer tracks. Peak axial and resultant cannon impact accelerations were 16–54% greater on the medium and hard tracks when compared to the soft tracks. Attenuation from hoof to cannon was not affected within the range of dirt track hardness tested.

**Conclusions:**

These results demonstrate that forelimb segmental accelerations are affected by track preparation manipulations within a single dirt track at galloping speeds. This may have implications for track preparation and injury risk management.

**Supplementary Information:**

The online version contains supplementary material available at 10.1186/s12917-026-05376-0.

## Background

Musculoskeletal injury is a major contributor to morbidity and mortality in equine athletes [3]. Catastrophic musculoskeletal injuries (CMI), which require euthanasia and occur at rates of 1–2 per 1000 starts, are of particular concern [6, 9]. The majority of CMIs are bone fractures or tendon ruptures thought to result from excessive loading of the limbs during high-speed gait [6, 16, 34, 36]. Repetitive, high magnitude impact loading has previously been associated with damage to bone, tendon, and cartilage [5, 21, 30, 34, 36]. Therefore, interventions to reduce and manage overloading of the limbs during training and racing are of great interest to reduce injury risk in equine athletes.

Track surface properties are modifiable factors that may influence musculoskeletal injury risk [9]; however, epidemiologic evidence is mixed. While some report lower rates of injury on synthetic tracks when compared to dirt [6, 20] or turf [6], others report higher rates on synthetic tracks when compared to turf [25, 37], or no difference between synthetic and turf tracks [4, 20]. The material properties of the track, manipulated through the composition, moisture content, cushion depth, and harrowing depth, may be the more relevant factors. Muddy and sloppy tracks have been associated with increased risk of injuries when compared to dry dirt and synthetic tracks [19]. Similarly, firmer surfaces have been associated with greater musculoskeletal injury risk [5, 24, 37]. Experiments using track testing devices have demonstrated greater peak forces on firmer track surfaces, supporting epidemiologic studies [15, 28].

Accelerometers have been used to characterize the impact phase of gait in equine athletes. Lower peak vertical hoof acceleration and peak ground reaction force have been observed on synthetic, harrowed dirt, and deeper tracks when compared to dirt, sealed dirt, turf, and shallow tracks, respectively ([11]; Setterbo et al., 2008; [15, 28]). On the other hand, peak vertical hoof acceleration was not associated with track hardness, measured using a trailer-mounted track testing device, when dirt track moisture content was varied [23]. Apart from Ratzlaff et al. [23], these studies used either track testing devices or only took measurements at trotting and cantering speeds. Additional work is needed to evaluate the influence of track properties at racing speeds. In addition to peak segmental accelerations, impact attenuation between segments may be calculated. The large impact acceleration peak observed at the hoof is attenuated by active (muscle) and passive (bone, tendon, cartilage) musculoskeletal structures in the hoof and limb [32]. This measure provides insight into the joint and soft tissue response to the impact phase of loading. The influence of track properties on impact attenuation has not yet been assessed. Therefore, the purpose of this study was to evaluate the effects of track hardness on segmental accelerations and impact attenuation in the equine forelimb at racing speeds. We hypothesized that harder surfaces would result in greater peak segmental accelerations and reduced attenuation.

## Methods 

### Animals

Twelve Thoroughbred chuckwagon outriding horses (geldings, ages 8–15 years, mass = 494.7 ± 30.7 kg) in active pre-season training were studied. Ten of the horses were shod in aluminum (*n* = 9) or steel (*n* = 1) racing plates and two of the horses were barefoot. All horses were deemed ‘fit to compete’ by their trainers and exhibited no observed gait abnormalities. Procedures were approved by the University of Calgary Animal Care Committee (AC21-0231 and AC23-0010) and written informed consent was obtained prior to testing by the owner.

### Track preparation and characterization

#### Track preparation

Testing was performed over two days at the Calgary Stampede racetrack. On each day the backstretch of the dirt racetrack was prepared in three lanes, clearly marked with pylons, where surface cushion depth was varied with decreasing depth from the inside to outside lanes. On the second day, a more compacted track underlay and lower harrowing depths were used to achieve harder track conditions. A high-speed video camera (120 Hz linear capture, GoPro HERO8, GoPro Inc., USA) was set-up along the inside rail at the middle of the data collection zone to evaluate lead/trailing limb side.

#### Track hardness and moisture measurements

Track hardness was measured at three locations along each lane using mechanical drop tests following modified American Society of Testing Materials standards F355 [1, 22, 35]. A uniaxial accelerometer (352C22 ICP accelerometer, ± 500 g range, PCB Piezotronics, USA) was attached to a cylindrical 9.1 kg mass with a 12.9 cm^2^ impacting surface. The mass was dropped through a guidance tube onto the surface from a height of 65.5 cm. Data were recorded at a sample rate of 1000 Hz using WinDAQ ADC (DataQ Instruments, USA). Moisture content of the track was measured three times at each of the same three sites along each lane using a soil moisture meter (MO750, Extech Instruments, USA). Track hardness and moisture tests were performed immediately prior to the morning sessions of the first and second testing days and immediately following the afternoon testing session on the first day (the surface was re-harrowed between the morning and afternoon sessions on the first day).

#### Data analysis

Acceleration data were normalized to baseline and converted from volts to g. The impact peak was identified for each trial and the mean of the three testing sites for each lane was calculated.

### Horse instrumentation and testing protocol

Tri-axial accelerometers (ADXL377, ± 200 g, 6.5 mV/g, Analog Devices Inc., USA) were attached at the lateral hoof and medial cannon of the left forelimb. The accelerometer at the hoof was attached with hot glue and stiff cloth tape. The accelerometer at the cannon was attached with double-sided tape and vet-wrap. The data acquisition box, which collected hoof and cannon acceleration data simultaneously at sample rate of 1600 Hz saved to a 2 GB SD card, was wrapped in bubble wrap and secured to the saddle with duct tape. A GPS-logger (10 Hz sample rate, recording on 8 GB SD card, Vbox Sport, Racelogic, USA) was attached to the back of the saddle, behind the seat, and used to record position and speed.

**Fig. 1:**
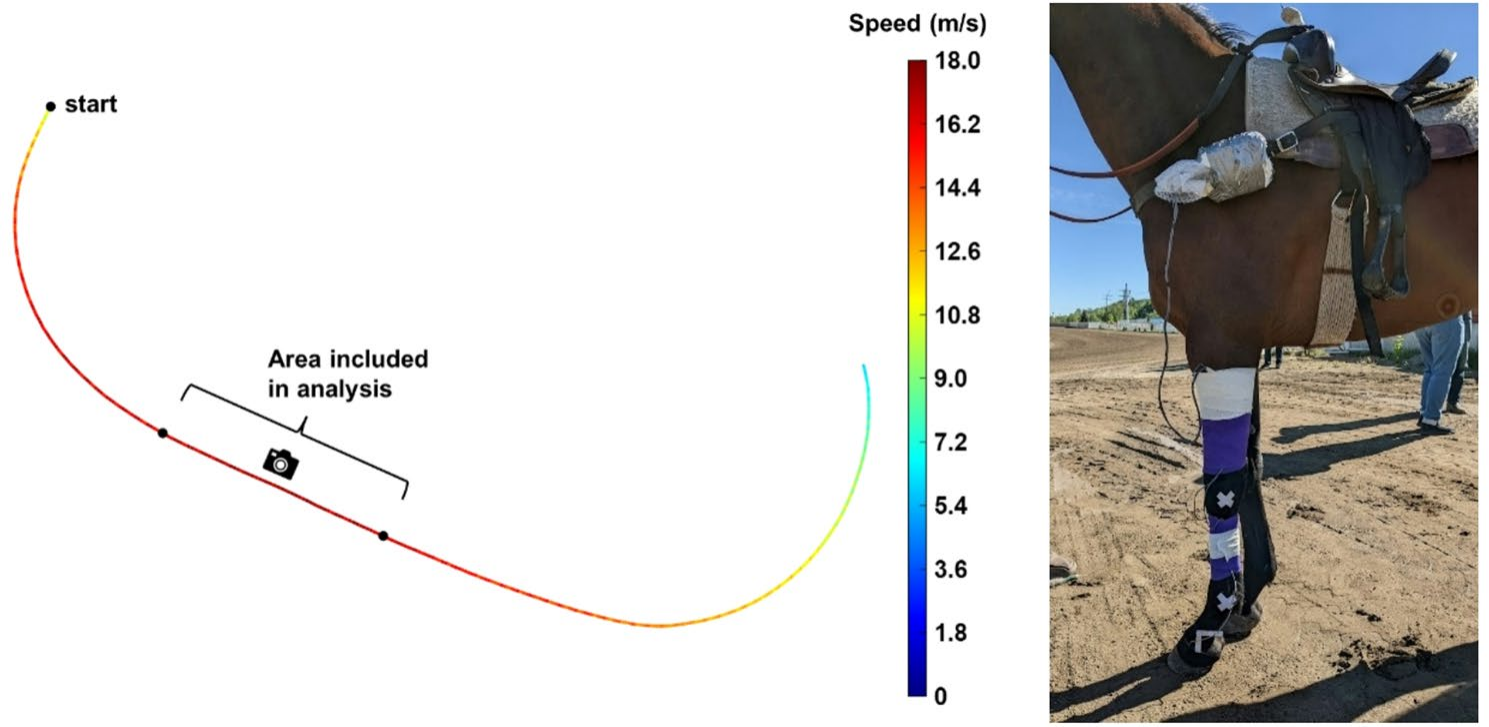
(Left) Example trial of horse speed along the track, measured by the GPS device (10 Hz). Horses started at the far turn then galloped along the backstretch. A GoPro camera was placed at approximately the middle of the data analysis area to determine whether the left leg was leading or trailing. (Right) Accelerometers were attached to the hoof and cannon. The data acquisition box was secured to the breast collar near the saddle. The GPS device was taped to the saddle behind the seat

Horses were ridden at a high-speed gallop along a 500 m section of the half-mile track starting at the far turn then straight along the backstretch (Fig. 1). The same jockey (male, 73.5 kg) performed all trials and aimed to achieve a speed of 16 m/s for each run by monitoring a GPS watch (fēnix 6, Garmin Ltd., USA). The speeds actually achieved were 15.5–17.9 m/s (mean = 17.3 m/s). Twelve horses were tested on the first day. Four of these horses were tested in the morning. The track was then reset by harrowing at the same depth as in the morning and additional water was added. The remaining eight horses were tested in the afternoon. On the second day, six of the horses returned. The testing protocol was repeated on three new track preparations.

### Data analysis – accelerations

#### Time domain

Hoof and cannon bone acceleration data were normalized to baseline (period of quiet standing prior to testing runs) and converted from volts to acceleration and normalized to gravitational acceleration. GPS data were synchronised to acceleration data using the systems’ timestamps. The approximate start of the horse’s run, beginning with a 180-degree turn was visually identified in the axial hoof acceleration. GPS coordinates corresponding to the start and end of the data collection zone in the backstretch were then used to cut the data. Acceleration and GPS speed were visually inspected to check the selected data. Mean GPS speed through the collection zone was calculated. Strides were detected using the axial hoof acceleration signal.

**Fig. 2:**
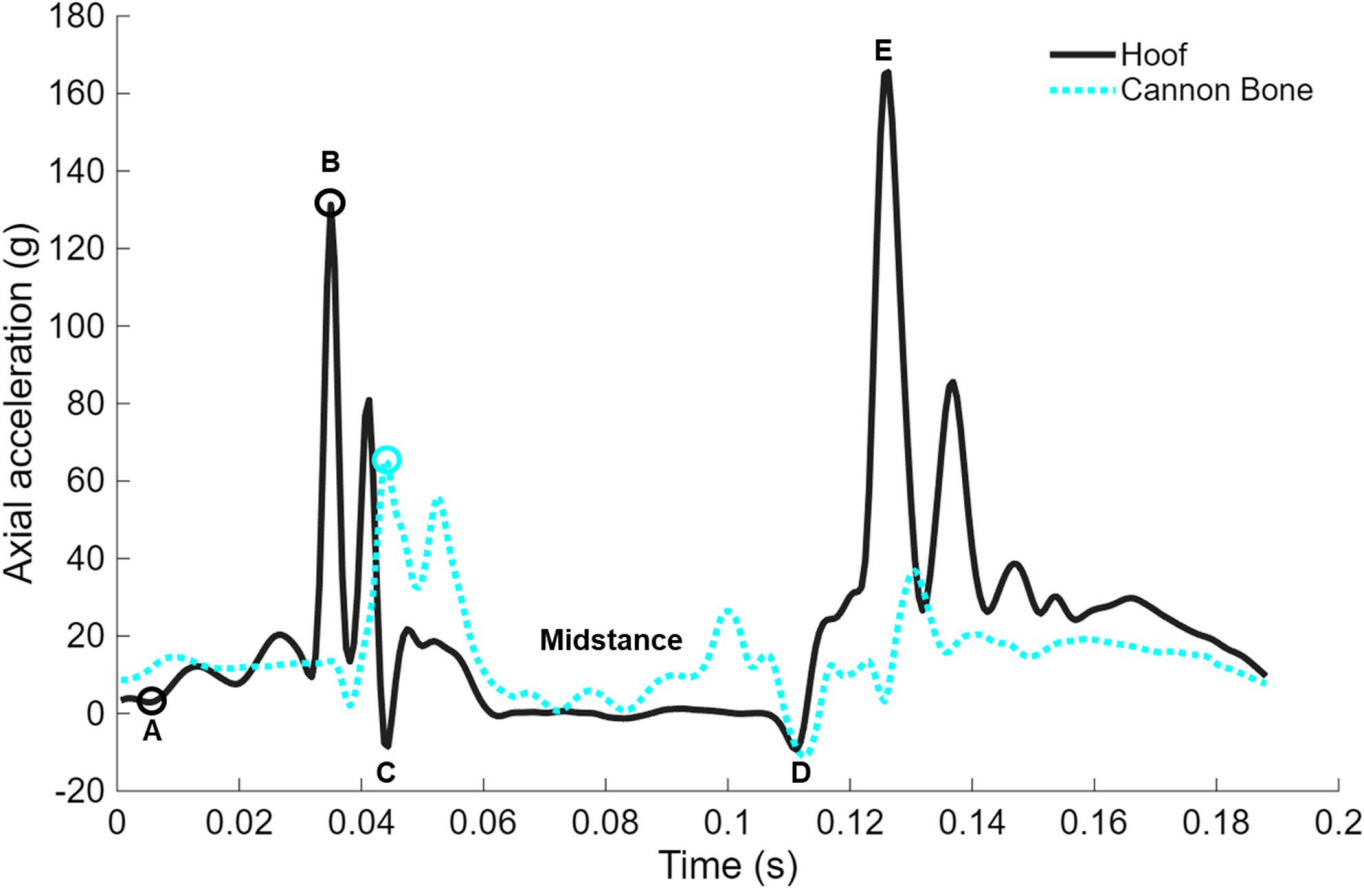
Exemplar axial hoof and cannon acceleration during stance. **A**: Hoof-ground contact. **B**: Impact peak. **C**: Shift from deceleration to propulsion. **D**: Hoof-off. **E**: large peak due to rotation associated with hoof-lift. The impact peaks for hoof and cannon acceleration signals are circled

For gait event detection, axial hoof acceleration was filtered using a low-pass Butterworth filter with a cut-off frequency of 10 Hz. Estimated stride time was calculated using an autocorrelation of the filtered axial hoof acceleration data, defined as the time difference between zero lag and the next largest peak. Acceleration peaks associated with specific gait events were then identified using a custom MATLAB algorithm (Mathworks Inc., USA). The largest peak, associated with the acceleration following hoof lift (Fig. 2, point E), within the first second of data in the collection zone was identified [23]. Subsequent hoof-lift peaks were identified as the largest acceleration value within 110% of the mean stride time. Working backwards from the hoof lift peaks, the small negative peak corresponding with hoof-off was identified (Fig. 2, point D). The negative peak associated with the shift from deceleration to propulsion was then identified (Fig. 2, point C). The hoof-ground impact peak was identified as the maximum peak between the preceding mid-swing phase and the propulsion peak (Fig. 2, point B). Finally, the approximate time of initial hoof-ground contact (Fig. 2, point A) was identified as the first time the acceleration signal value was below 3 g, working backward from the impact peak up to 0.100 s. If the signal did not go below 3 g, the time of the minimum signal value in the same window was used as the initial hoof-ground contact point.

Resultant acceleration was calculated. Axial and resultant signals were then filtered using a 4th order Butterworth filter with a low-pass cut-off frequency of 250 Hz, which was the mean frequency that retained 98% of the axial hoof acceleration signal power as determined from spectral analysis. Peak axial and resultant impact accelerations at the hoof and cannon were identified within a search window from hoof-ground contact to 0.030 s after the impact peak identified in the smoothed axial hoof signal. For post-hoc assessments of the hoof dorso-palmar and medial-lateral accelerations, the signals were filtered in the same manner as the axial acceleration then peaks were identified as the maximum value in a search window from hoof-ground contact to 0.030 s after the shift point from deceleration to propulsion. Impact attenuation in the time domain was calculated using the equation:

$$\:Attenuation=\:\left(1-\frac{{peak\:axial\:acceleration}_{cannon}}{{peak\:axial\:acceleration}_{hoof}}\right)x100$$

where attenuation is expressed as a percentage; positive values indicate attenuation and negative values indicate gain.

#### Frequency domain

Frequency domain signal power and impact attenuation were calculated from the power spectral density curve with a frequency bin resolution of 1 Hz. A 0.200 s window encompassing part of the swing phase, the hoof-ground impact and subsequent stance phase up to 0.003 s prior to hoof-off was analyzed. Signal power magnitude was calculated as the area under the power spectral density curve within the frequency band of 11–20 Hz. This frequency band represents the second major component of the cannon power spectral density curve, corresponding to the frequency components due to impact (Greco-Otto et al., 2019). For impact attenuation, a transfer function was calculated as.

$$\:Transfer\:Function=10\mathrm{log}10\left({PSD}_{cannon}/{PSD}_{hoof}\right)$$

where PSD is the power spectral density of the axial acceleration signal. The resulting values were converted to a linear scale, averaged within the 11–20 Hz band, then converted back to decibels. The resulting measure of impact attenuation represents a gain (positive) or attenuation (negative) in signal power between the hoof and cannon.

**Fig. 3:**
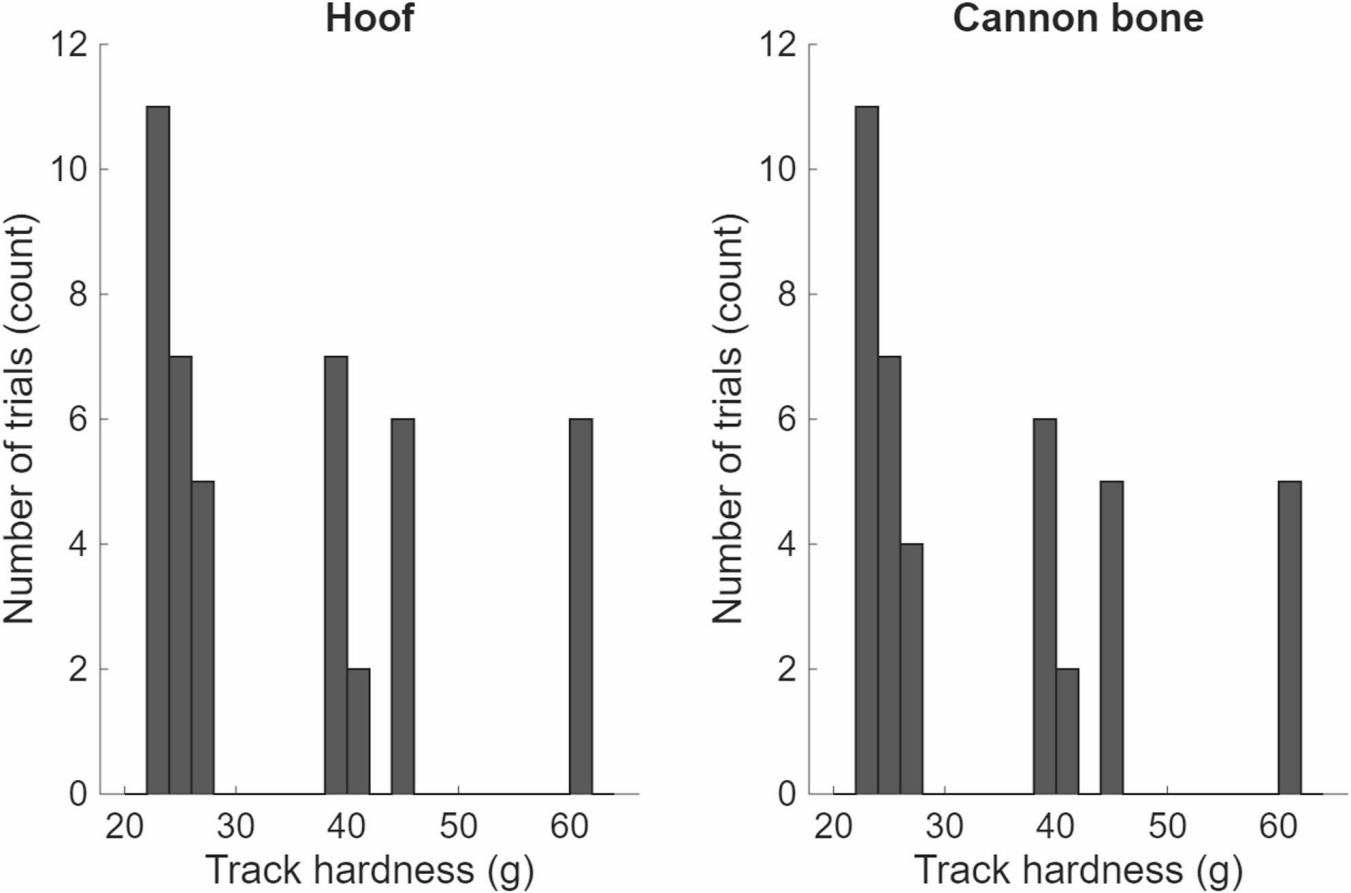
Distribution of trials collected across tracks with varying hardness. Track hardness clustered into three categories: soft (22.3–26.3 g, 22–23 trials), medium (38.1–44.3 g, 13–15 trials) and hard (61.2 g, 5–6 trials). Due to equipment malfunction during testing resulting in obvious artefacts in the cannon acceleration data, four of the trials for the cannon and attenuation outcomes were excluded from the analysis

### Statistics

Track hardness across sessions (Day 1 morning, Day 1 afternoon, Day 2) was observed to cluster into three distinct groups (Fig. 3). As a result, we pooled the data from the tracks into soft (22.3–26.3 g, 22–23 trials from 11 horses), medium (38.1–44.3 g, 13–15 trials from 10 to 11 horses) and hard (61.2 g, 5–6 trials from 5 to 6 horses) groups. The varying number of trials in each category results from multiple tracks for some horses within the binned categories as well as missing trials due to technical failures of the equipment. Data for three of the horses on the first day were not recorded or contained significant gaps or obvious artefacts in the data, which excluded them from analysis. Details of the trials included in each track category are provided in the Supplementary Material (Table S1).

The mean peak segmental accelerations, time-domain impact attenuation, signal power magnitude, and frequency-domain impact attenuation for ten strides were calculated. Linear mixed models were used to explore the effect of track hardness on peak segmental accelerations, signal power magnitudes, and impact attenuations, with soil moisture and speed as covariates. Track hardness category, speed, and moisture were set as fixed effects. Horse was set as a random effect. If moisture was not significant, it was removed from the model. Speed was kept in the models. Post-hoc pairwise comparisons were assessed using t-tests with p-values adjusted for multiple comparisons using the Tukey method. All statistical analyses were completed using R (v4.5.0, lmerTest: [13]; emmeans: [14]).

## Results

### Track properties

Track hardness varied between lane preparations from 22.3 to 61.2 g and moisture content varied from 12.1 to 27.4%. Track hardness of the lanes across sessions clustered into three distinct groups: soft (mean = 23.9 g, range = 22.3–26.3 g), medium (mean = 41.2 g, range = 38.1–44.3 g), and one hard (61.2 g) track. Soil moisture content varied within soft (17.8–27.4%) and medium (12.6–21.8%) hardness tracks and was the lowest in the hard track (12.1%).

### Time domain

Mean (range) speed through the collection zone was 16.4 (15.4 – 17.9) m/s on the soft tracks, 16.5 (15.3 – 17.5) m/s on the medium tracks, and 17.2 (16.7 – 17.6) m/s on the hard track. As mean speed was higher on the hard track when compared to the soft and medium tracks (*p* = 0.0028 and 0.0078, respectively), it was kept as a covariate in the regression models.

**Fig. 4:**
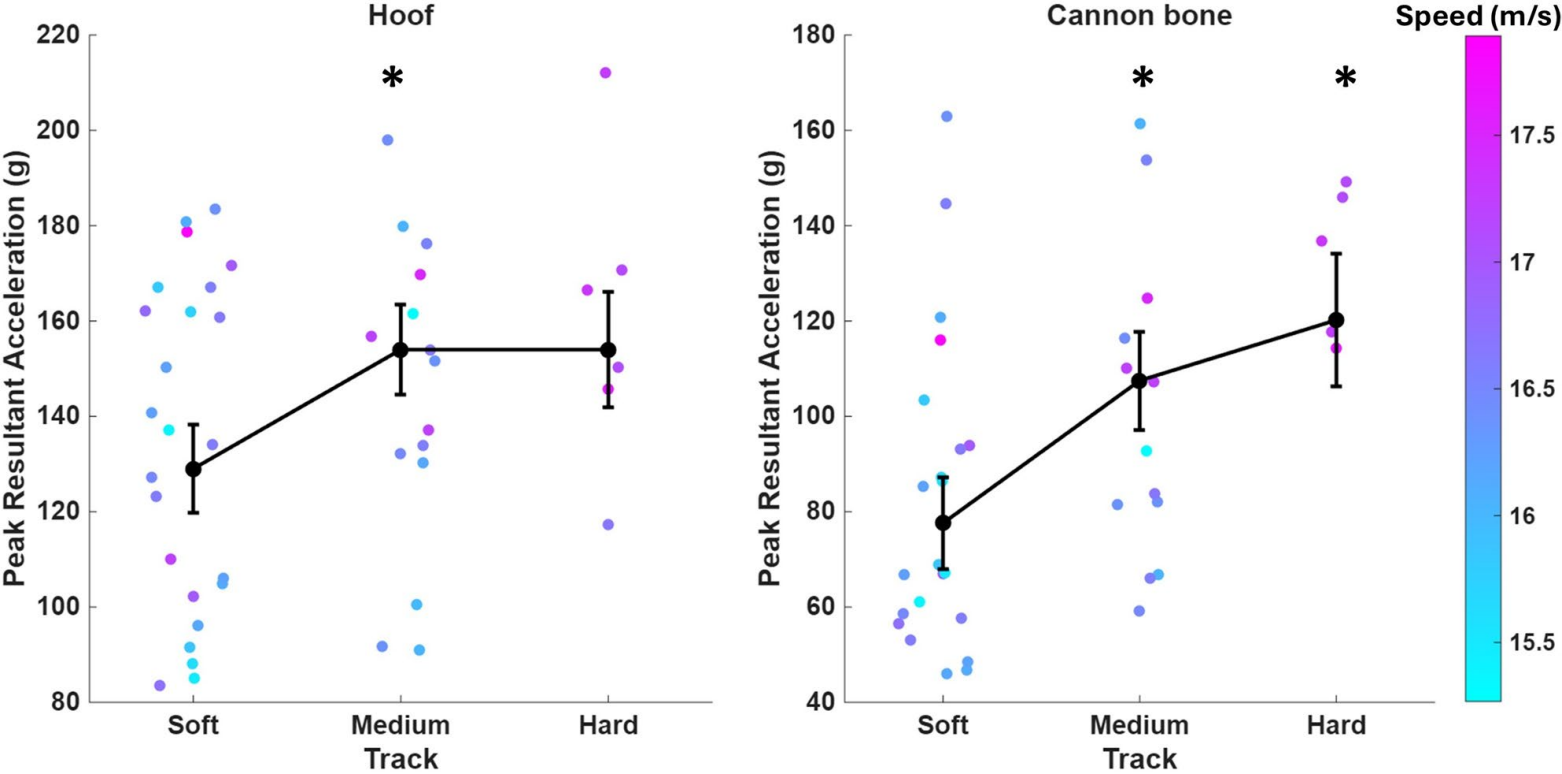
Peak resultant hoof (left) and cannon (right) acceleration across soft (23.9 g), medium (41.2 g), and hard (61.7 g) track hardness categories. Colored dots represent mean peak accelerations for each horse within a trial, where the color indicates speed. The black dots represent the estimated marginal means (and standard errors) calculated from the linear mixed models that accounted for speed, moisture, and horse variables. * significantly different when compared to the soft track

Significant main effects of track hardness were observed for peak axial and resultant cannon bone acceleration (*p* = 0.0013 and *p* = 0.0060), and for peak resultant hoof acceleration (*p* = 0.0402). Mean (SE) peak axial cannon bone acceleration was 8.6 (2.4) g and 14.6 (3.6) g greater on the medium and hard tracks when compared to the soft tracks (*p* = 0.0036 and *p* = 0.0012), respectively. Mean (SE) peak resultant cannon bone acceleration was 29.9 (9.7) g and 42.6 (14.3) g greater on the medium and hard tracks when compared to the soft tracks (*p* = 0.0104 and *p* = 0.0166, Fig. 4), respectively. Peak resultant hoof acceleration was greater on the medium tracks when compared to the soft tracks (difference = 25.5 (8.0) g, *p* = 0.0093, Fig. 4). No differences in acceleration measures were observed between the medium and hard tracks (*p* > 0.05).

Main effects were observed for speed in the axial hoof (coefficient (SE) = 13.1 (5.0), *p* = 0.0131), resultant hoof (coefficient (SE) = 18.0 (6.2), *p* = 0.0063), and axial cannon bone (coefficient (SE) = 8.0 (1.9), *p* = 0.0001) acceleration models. Main effects for moisture were observed for resultant hoof (coefficient (SE) = 2.49 (0.79), *p* = 0.0036), axial cannon bone (coefficient (SE) = 0.65 (0.22), *p* = 0.0075), and resultant cannon bone (coefficient (SE) = 2.40 (0.88), *p* = 0.0113) acceleration models. No predictors were significant for hoof-cannon bone time-domain attenuation (*p* > 0.05). Detailed model results are available in the Supplementary Material.

### Frequency domain

In the frequency domain, significant main effects of track hardness were observed for resultant cannon bone signal power magnitude (medium track *p* = 0.0036 and hard track *p* = 0.0005). Mean (SE) resultant cannon bone signal power magnitude was 49.4 (15.4) g^2^/Hz and 93.3 (23.5) g^2^/Hz greater on the medium and hard track surfaces when compared to the soft track surfaces (*p* = 0.0097 and *p* = 0.0014), respectively. Track surface was also a significant predictor in the hoof-cannon bone frequency domain attenuation model (coefficient = -1.14 (0.48), *p* = 0.0265). However, no pairwise comparisons between track hardness groups were significant (*p* > 0.05). Main effects were observed for speed in the resultant hoof (coefficient (SE) = 35.4 (8.1), *p* = 0.0001), and axial cannon bone (coefficient (SE) = 10.0 (3.8) g) signal power magnitude models (*p* = 0.0119). A main effect of moisture was observed for resultant cannon (coefficient (SE) = 3.17 (1.44) g) signal power magnitude models (*p* = 0.0369).

## Discussion

The purpose of this study was to evaluate the effects of track hardness on segmental accelerations and impact attenuation in the equine forelimb at racing speeds (15.2–17.9 m/s in this study). We hypothesized that harder surfaces would result in greater peak segmental accelerations and reduced attenuation. Our hypothesis was partially confirmed: greater peak segmental accelerations were observed on the medium and hard track surfaces when compared to the softer track surfaces. On the other hand, hoof-cannon bone attenuation in both the time and frequency domains was not affected by track hardness. These results demonstrate the influence of dirt track properties on forelimb hoof and cannon accelerations and may have practical implications for injury risk management as track preparation is a readily modifiable factor.

Peak axial and resultant cannon bone, and resultant hoof accelerations significantly increased with track hardness. These results are consistent with data from track testing devices simulating hoof impact [15, 28]. A stiffer surface deforms less during impact, reducing the time and distance over which the limb decelerates at hoof-track impact, increasing peak accelerations and forces. While resultant hoof acceleration was not statistically different between the soft and hard tracks, it is likely that the result is a consequence of the small sample size on the hard track (Fig. 4). Similarly, the small sample size on the hard track could explain why no difference was detected between the medium and hard tracks for resultant hoof and cannon bone peak accelerations. Alternatively, the horses may have made kinematic adjustments on the hard surface to limit further increases in impact accelerations. Future work could use optical or IMU-based motion capture to assess kinematics in response to track hardness.

We observed no change in time-domain impact attenuation between the hoof and cannon bone between track hardness conditions, suggesting that the high impacts are not being absorbed by soft tissues at the joints. Given the short period of impact and the main muscles being in the proximal limb, the horse may have limited ability to adjust fetlock joint kinematics and stiffness to change active or passive attenuation [18].

Interestingly, we observed no differences in peak axial hoof acceleration at impact across tracks of varying hardness, similar to Ratzlaff et al.,’s [23] experiment at galloping speeds. However, peak resultant hoof acceleration measured in this study was on average 19% greater on the medium hardness tracks when compared to the softer tracks. Accelerations were not statistically greater on the hardest surface, but this was likely due to the smaller sample size in this condition resulting in greater standard error (Fig. 4). The difference observed between medium and soft surfaces for resultant but not axial hoof accelerations suggests differences in horizontal-plane accelerations. Post-hoc analysis of the dorso-palmar and medial-lateral impact peaks (see Supplementary Material) confirmed this, showing greater acceleration peaks in both dorso-palmar and medial-lateral directions on the medium tracks when compared to the soft tracks (see Supplemental Material). The greater dorso-palmar and medial-lateral acceleration peaks on the medium tracks may suggest greater shear stresses at the hoof when compared to the soft surfaces [7, 32]. Experimental tests using a track testing device observed a positive correlation between cushion depth and shear stress [26] however, a correlation between segmental accelerations and shear stress in response to track manipulation has not yet been tested.

In the frequency domain, resultant cannon bone signal power magnitude was the only acceleration measure influenced by track hardness. Like the time domain results, resultant cannon bone acceleration signal power magnitude was greater on medium and hard tracks when compared to soft tracks, by 59% and 111%, respectively. In contrast, track hardness did not influence axial hoof, axial cannon bone, nor resultant hoof acceleration signal power magnitudes. Signal power magnitude was calculated using a 0.200 s data window that included part of the swing phase and the entire hoof-ground contact up to just before hoof-off. While only the frequency band associated with impact was assessed, it is possible that other peaks in the hoof and axial cannon bone acceleration curves, such as the peaks associated with the transition from breaking to propulsion (Fig. 2), behaved differently in response to track hardness and introduced additional variation in the data.

While this study investigated a single racetrack, we demonstrate here that within dirt track preparation manipulations can significantly influence limb impact accelerations at galloping speeds. This may have implications for track maintenance and injury risk management efforts as surface hardness, manipulated through factors including harrowing depth and underlay compaction, are easily modifiable. Harder surfaces, which in this study increased segmental accelerations and speed, have been associated with greater rates of musculoskeletal injury [2, 5, 24, 37]. Thus, a softer surface achieved through a less compacted track underlay and/or greater harrowing depth may be beneficial. Regular testing of track hardness may also be warranted to enable further site-specific and across-site assessments of the potential link between dirt track hardness and injury risk. However, additional properties of the track surface or trade-offs may also need to be considered. The greater horizontal acceleration peaks observed in this study on the harder surfaces could indicate increased off-axis loading or perhaps higher shear forces. Conversely, the greater horizontal peaks may also indicate less hoof-slip, which could decrease injury risk [8, 26]. Furthermore, softer surfaces may require more frequent maintenance due to faster compaction which increases surface hardness and the variability in hardness across the track [12, 28]. Finally, it is important to note that there are limitations in inferring musculoskeletal loading and injury risk from impact accelerations alone [33]. Setterbo et al. [27] observed similar changes between dirt, turf, and synthetic surfaces for both impact acceleration and active (midstance) peak force at the hoof. However, load magnitudes are lower during the impact phase when compared to mid-stance and these two peaks may not necessarily be correlated with each other or with peak impact accelerations due to kinematic adjustments between the impact and midstance timepoints [17, 32]. For example, the accelerations observed at impact are a function of both impact velocity and segment orientations/joint angles which influence accelerations via changes in effective mass [29]. That said, there are challenges with measuring segment orientations and modeling musculoskeletal loads, especially at racing speeds. Instrumented horseshoes are the most feasible method to measure force during high-speed gaits in the field, but deformation of the shoe may produce errors [32]. Additionally, instrumented horseshoes are thicker and heavier than racing or training shoes and may influence the hoof-surface interaction [27]. Calculating joint kinetics also requires measurement of kinematics (i.e., limb joint angles and hoof-ground contact angle). Tracking hoof movement as the hoof sinks into the surface requires use of purpose-built extension bars that are rigidly affixed to the hoof to position the reflective markers above where the hoof would be submerged [10, 31]. Furthermore, large outdoor data collection areas remain a challenge for motion capture approaches. Inertial measurement units may be useful to tackle this challenge. Thus, further research is warranted to better understand the influence of modifiable racetrack properties on limb loading, impact accelerations, and injury risk in racehorses.

Another limitation of this study was the small sample size, particularly on the hardest surface condition. As previously discussed, the small sample size in the hard track condition may explain why no statistically significant differences were observed for peak resultant hoof acceleration between the hard and soft tracks (Fig. 4). Due to our limited power, we also chose to not account for whether the instrumented forelimb was the lead or trailing limb. It has been suggested that the leading limbs may experience greater peak impact accelerations [11] although another experiment observed no difference between lead and trailing forelimbs [23]. Injury risk data for lead and trailing limbs is also mixed [20]. In our study, the left forelimb was the lead leg in 50–67% of trials within each track hardness category. Raw data does not indicate differences in peak segmental accelerations or attenuation (in time or frequency domains) between the lead ant trailing leg trials except perhaps for the axial hoof acceleration measures (Supplemental Fig. 1), although this could also be an artefact of the small sample size or that the visualized raw data has not been adjusted for speed.

## Conclusions

In summary, we observed 16% – 55% greater peak cannon accelerations and 19% greater peak resultant hoof accelerations at impact on medium and hard tracks when compared to softer tracks. Our results demonstrate that surface preparation manipulations can significantly influence limb accelerations at galloping speeds. This may have implications for injury risk management; track hardness, varied in our study through harrowing and moisture content, are modifiable factors. Further work is needed to evaluate the effect of these parameters on injury risk and ideal hardness ranges.

## Supplementary information

Supplementary Material 1.

## Data Availability

The datasets used and/or analysed during the current study are available from the corresponding author on reasonable request.
