# Supplementary material for: The influence of dirt track hardness on equine limb acceleration and impact attenuation
Source: BMC Vet Res. 2026 Mar 19;22:251. doi: 10.1186/s12917-026-05376-0 (PMC13123126; doi:10.1186/s12917-026-05376-0)
Supplement: Supplementary file 1 — Supplementary Material 1. [file 12917_2026_5376_MOESM1_ESM.pdf]

Supplementary Materials

The Influence of Dirt Track Hardness on Equine Limb Acceleration and Impact Attenuation

Olivia L Bruce[a,b,e*], Thilo Pfau[b,c,d], Laura E. Crack[b,d], Andrew Sawatsky[b,d], Renaud Leguillette[c] , W Brent Edwards[a,b,d]

[a] Department of Biomedical Engineering, Schulich School of Engineering, University of Calgary, Calgary, AB, Canada

[b] McCaig Institute for Bone and Joint Health, Cumming School of Medicine, University of Calgary, Calgary, AB, Canada

[c] Faculty of Veterinary Medicine, University of Calgary, Calgary, AB, Canada

[d] Faculty of Kinesiology, University of Calgary, Calgary, AB, Canada

[e] Department of Radiology, Stanford University, Stanford, CA, USA

*Corresponding author:

Olivia L Bruce

1201 Welch Rd, Rm P093, Stanford University

Stanford, CA 94305 USA

Email: obruce@stanford.edu

Horse Trial Details

The soft track category included 3 testing lanes over the two days. There were 24 trials included from 11 horses. (4 horses ran on all 3 soft lanes, 5 horses ran on 2 of the soft lanes).

The medium track category included 2 testing lanes over the two days. There were 15 trials included from 11 horses (4 horses ran on both tracks).

The hard track category included only one lane on the second day. 6 trials were included from 6 horses.

Table S1: Breakdown of the trials included in each track category by session and horse

Track	Track category	Number of trials	Participants
Day 1, Lane 1	Soft	9 (3 in am session, 6 in pm session)	am: 2, 3, 4<br>pm: 6, 7, 8, 9, 10, 12
Day 1, Lane 2	Soft	9 (3 in am session, 6 in pm session)	am: 2, 3, 4<br>pm: 6, 7, 8, 9, 10, 12
Day 1, Lane 3	Medium	9 (3 in am session, 6 in pm session)	am: 2, 3, 4<br>pm: 6 (no cannon), 7, 8, 9, 10, 12
Day 2, Lane 1	Soft	6	1, 2 (no cannon), 5, 7 (no cannon), 10, 12
Day 2, Lane 2	Medium	6	1, 2 (no cannon), 5, 7, 10, 12
Day 2, Lane 3	Hard	6	1, 2 (no cannon), 5, 7, 10, 12

* “no cannon”: artefacts in the data due to equipment issues resulted in no cannon acceleration data for these participants. The hoof variables for these trials were still included.

Horses 5 and 11 performed trials on Day 1, but equipment failures resulted in no usable data.

Shoe types:

Aluminium: Horses 1, 2, 4, 6, 7, 8, 9, 10, 12

Barefoot: Horses 5, 11

Steel: Horse 3

Lead leg

Figure S1: Mean peak and resultant accelerations and hoof-cannon attenuation across tracks, comparing between lead leg (magenta) and trailing leg (cyan).

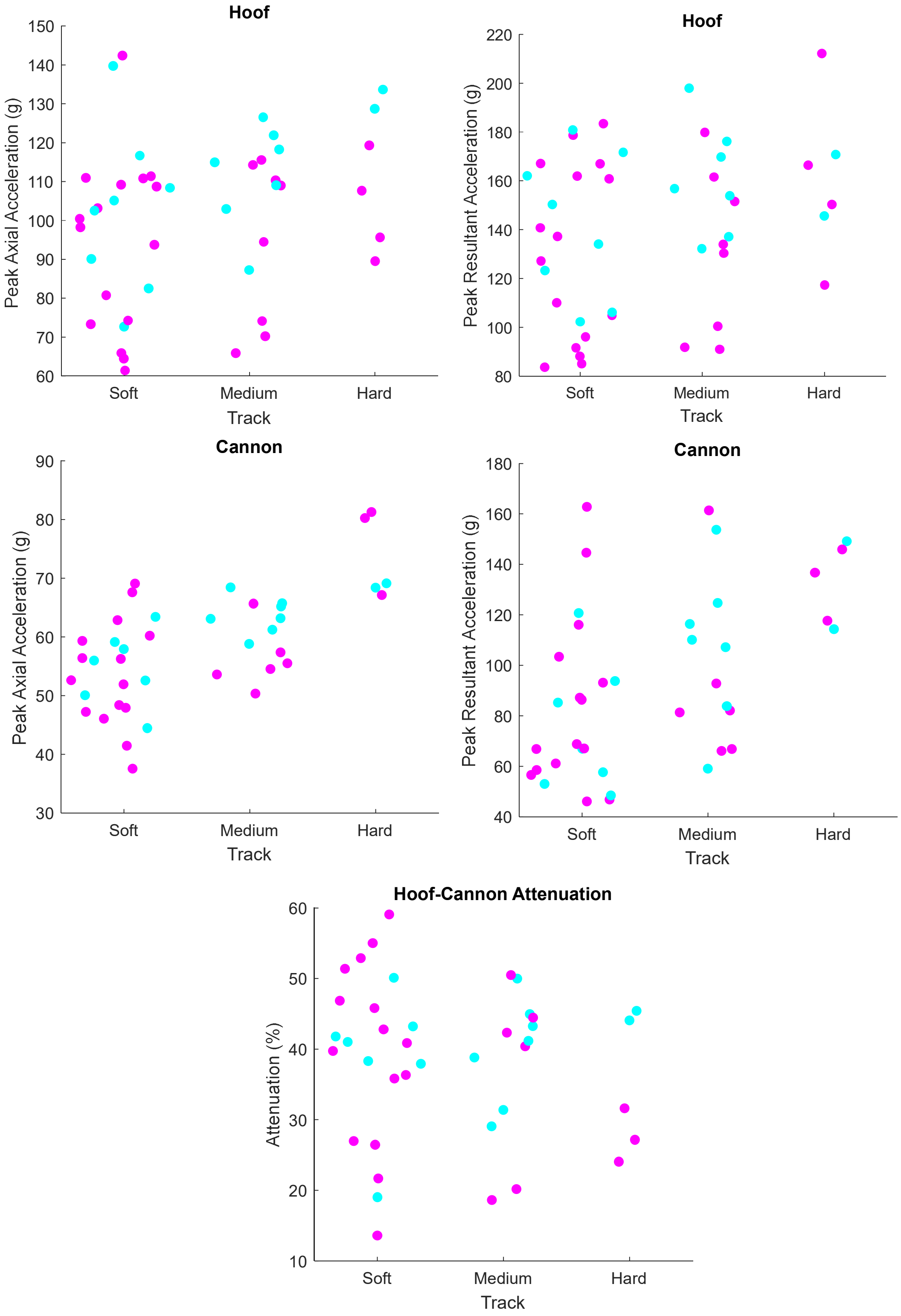

Statistical model outputs

Time Domain

Peak axial hoof acceleration

Linear mixed model fit by REML. t-tests use Satterthwaite's method ['lmerModLmerTest']

Formula: Hoof acceleration ~ track category + speed + moisture + (1 | Horse)

```
REML criterion at convergence: 351.7

Scaled residuals:
    Min      1Q  Median      3Q     Max
-2.2740 -0.4479 -0.0059  0.5149  2.4175

Random effects:
 Groups   Name        Variance Std.Dev.
 Horse    (Intercept) 165.5    12.86
 Residual             177.8    13.33
Number of obs: 45, groups:  Horse, 11

Fixed effects:
             Estimate Std. Error       df t value Pr(>|t|)
(Intercept) -143.4868    80.9990  38.0410  -1.771   0.0845 .
TrackCat2     13.1564     6.5710  31.1529   2.002   0.0540 .
TrackCat3     14.4281     9.5715  32.7739   1.507   0.1413
speed         13.0121     4.8696  37.9716   2.672   0.0110 *
moisture       1.2427     0.6442  31.2191   1.929   0.0628 .
---
Signif. codes:  0 ‘***’ 0.001 ‘**’ 0.01 ‘*’ 0.05 ‘.’ 0.1 ‘ ’ 1

Correlation of Fixed Effects:
          (Intr) TrckC2 TrckC3 speed
TrackCat2 -0.083
TrackCat3  0.268  0.622
speed     -0.982 -0.064 -0.397
moisture  -0.172  0.740  0.636 -0.006
```

Moisture removed

Formula: Hoof acceleration ~ track category + speed + (1|Horse)

```
REML criterion at convergence: 356.3

Scaled residuals:
     Min       1Q   Median       3Q      Max
-2.54313 -0.49480  0.02417  0.46469  2.25728

Random effects:
 Groups   Name        Variance Std.Dev.
 Horse    (Intercept) 166.6    12.91
 Residual             192.0    13.86
Number of obs: 45, groups:  Horse, 11

Fixed effects:
            Estimate Std. Error       df t value Pr(>|t|)
(Intercept) -117.269     82.586   39.496  -1.420   0.1635
TrackCat2      3.783      4.594   31.900   0.823   0.4164
TrackCat3      2.699      7.667   34.763   0.352   0.7270
speed         13.106      5.041   39.425   2.600   0.0131 *
---
Signif. codes:  0 ‘***’ 0.001 ‘**’ 0.01 ‘*’ 0.05 ‘.’ 0.1 ‘ ’ 1

Correlation of Fixed Effects:
          (Intr) TrckC2 TrckC3
TrackCat2  0.067
TrackCat3  0.496  0.292
speed     -0.998 -0.088 -0.510
```

Peak axial cannon acceleration

Linear mixed model fit by REML. t-tests use Satterthwaite's method ['lmerModLmerTest']

Formula: Cannon acceleration ~ track category + speed + moisture + (1 | Horse)

```
REML criterion at convergence: 235.4

Scaled residuals:
     Min       1Q   Median       3Q      Max 
-1.83097 -0.49539  0.07057  0.39846  1.76816 

Random effects:
 Groups   Name        Variance Std.Dev.
 Horse    (Intercept) 23.68    4.866   
 Residual             20.09    4.482   
Number of obs: 40, groups:  Horse, 11

Fixed effects:
            Estimate Std. Error       df t value Pr(>|t|)    
(Intercept) -90.9970    30.8365  32.6165  -2.951 0.005826 ** 
TrackCat2     8.6412     2.3850  24.9072   3.623 0.001300 ** 
TrackCat3    14.6366     3.5923  26.5593   4.074 0.000373 ***
speed         8.0273     1.8667  32.4243   4.300 0.000146 ***
moisture      0.6495     0.2231  24.9195   2.912 0.007476 ** 
---
Signif. codes:  0 ‘***’ 0.001 ‘**’ 0.01 ‘*’ 0.05 ‘.’ 0.1 ‘ ’ 1

Correlation of Fixed Effects:
          (Intr) TrckC2 TrckC3 speed 
TrackCat2  0.017                     
TrackCat3  0.356  0.628              
speed     -0.985 -0.149 -0.467       
moisture  -0.153  0.728  0.594 -0.009
```

Marginal means

```
$emmeans
 TrackCat emmean   SE   df lower.CL upper.CL
 1          53.6 1.98 18.4     49.5     57.8
 2          62.3 2.16 23.4     57.8     66.7
 3          68.3 3.21 34.9     61.7     74.8

Degrees-of-freedom method: kenward-roger 
Confidence level used: 0.95 

$contrasts
 contrast              estimate   SE   df t.ratio p.value
 TrackCat1 - TrackCat2    -8.64 2.39 25.8  -3.608  0.0036
 TrackCat1 - TrackCat3   -14.64 3.64 27.4  -4.024  0.0012
 TrackCat2 - TrackCat3    -6.00 2.83 27.1  -2.119  0.1047

Degrees-of-freedom method: kenward-roger 
P value adjustment: tukey method for comparing a family of 3 estimates
```

Attenuation between hoof and cannon

Linear mixed model fit by REML. t-tests use Satterthwaite's method ['lmerModLmerTest']
Formula: attenuation ~ track category + speed + moisture + (1 | Horse)

```
REML criterion at convergence: 277.6

Scaled residuals:
     Min       1Q   Median       3Q      Max 
-2.55911 -0.50654  0.00926  0.53297  1.64289 

Random effects:
 Groups   Name        Variance Std.Dev.
 Horse    (Intercept) 71.42    8.451   
 Residual             69.06    8.310   
Number of obs: 40, groups:  Horse, 11

Fixed effects:
            Estimate Std. Error       df t value Pr(>|t|)
(Intercept) 21.60010   56.66317 33.31548   0.381    0.705
TrackCat2   -0.73855    4.41867 25.22187  -0.167    0.869
TrackCat3   -6.53152    6.64468 27.02894  -0.983    0.334
speed        1.00656    3.43097 33.15343   0.293    0.771
moisture     0.04449    0.41327 25.23058   0.108    0.915

Correlation of Fixed Effects:
          (Intr) TrckC2 TrckC3 speed 
TrackCat2  0.016                     
TrackCat3  0.355  0.628              
speed     -0.985 -0.150 -0.467       
moisture  -0.152  0.728  0.595 -0.011
```

Remove moisture from model

Formula: attenuation ~ track category + speed + (1 | Horse)

```
REML criterion at convergence: 277.7

Scaled residuals:
     Min       1Q   Median       3Q      Max 
-2.63355 -0.52185 -0.00373  0.55741  1.67648 

Random effects:
 Groups   Name        Variance Std.Dev.
 Horse    (Intercept) 72.62    8.522   
 Residual             66.35    8.145   
Number of obs: 40, groups:  Horse, 11

Fixed effects:
            Estimate Std. Error      df t value Pr(>|t|)
(Intercept)  23.4056    55.1101 34.1535   0.425    0.674
TrackCat2    -1.0628     2.9692 26.3566  -0.358    0.723
TrackCat3    -6.9144     5.2441 28.9273  -1.318    0.198
speed         0.9566     3.3759 34.0797   0.283    0.779

Correlation of Fixed Effects:
          (Intr) TrckC2 TrckC3
TrackCat2  0.188              
TrackCat3  0.561  0.354       
speed     -0.998 -0.207 -0.573
```

Peak resultant hoof acceleration

Linear mixed model fit by REML. t-tests use Satterthwaite's method ['lmerModLmerTest']
Formula: resultant hoof acceleration ~ track category + speed + moisture + (1 | Horse)

```
REML criterion at convergence: 376.2

Scaled residuals:
     Min       1Q   Median       3Q      Max
-2.30529 -0.53940 -0.05724  0.58249  1.70818

Random effects:
 Groups   Name        Variance Std.Dev.
 Horse    (Intercept) 692.7    26.32
 Residual             264.1    16.25
Number of obs: 45, groups:  Horse, 11

Fixed effects:
             Estimate Std. Error       df t value Pr(>|t|)
(Intercept) -214.8074   103.0978  33.9765  -2.084  0.04480 *
TrackCat2     25.5335     8.0307  30.4129   3.179  0.00338 **
TrackCat3     25.2521    11.7954  31.1238   2.141  0.04023 *
speed         18.0252     6.1880  33.6374   2.913  0.00632 **
moisture       2.4872     0.7875  30.4421   3.158  0.00357 **
---
Signif. codes:  0 ‘***’ 0.001 ‘**’ 0.01 ‘*’ 0.05 ‘.’ 0.1 ‘ ’ 1

Correlation of Fixed Effects:
          (Intr) TrckC2 TrckC3 speed
TrackCat2 -0.081
TrackCat3  0.276  0.622
speed     -0.982 -0.060 -0.401
moisture  -0.171  0.741  0.632  0.001
```

Estimated marginal means

```
$emmeans
 TrackCat emmean    SE   df lower.CL upper.CL
 1           129  9.18 14.2      109      149
 2           154  9.51 16.2      134      175
 3           154 12.20 30.9      129      179

Degrees-of-freedom method: kenward-roger
Confidence level used: 0.95

$contrasts
 contrast              estimate    SE   df t.ratio p.value
 TrackCat1 - TrackCat2  -25.534  8.04 30.2  -3.176  0.0093
 TrackCat1 - TrackCat3  -25.252 11.80 31.0  -2.132  0.0998
 TrackCat2 - TrackCat3    0.281  9.30 31.0   0.030  0.9995

Degrees-of-freedom method: kenward-roger
P value adjustment: tukey method for comparing a family of 3 estimates
```

Peak resultant cannon acceleration

Linear mixed model fit by REML. t-tests use Satterthwaite's method ['lmerModLmerTest']
Formula: resultant cannon acceleration ~ track category + speed + moisture + (1 | Horse)

```
REML criterion at convergence: 336.9

Scaled residuals:
     Min       1Q   Median       3Q      Max
-1.30003 -0.73736 -0.03821  0.45658  1.77306

Random effects:
 Groups   Name        Variance Std.Dev.
 Horse    (Intercept) 739.8    27.20
 Residual             308.5    17.56
Number of obs: 40, groups:  Horse, 11

Fixed effects:
             Estimate Std. Error       df t value Pr(>|t|)
(Intercept) -107.7170   125.5822  29.5531  -0.858  0.39794
TrackCat2     29.9028     9.3780  25.0013   3.189  0.00382 **
TrackCat3     42.5777    14.2224  25.8709   2.994  0.00600 **
speed          8.5094     7.5902  29.2587   1.121  0.27136
moisture       2.4001     0.8772  25.0180   2.736  0.01127 *
---
Signif. codes:  0 ‘***’ 0.001 ‘**’ 0.01 ‘*’ 0.05 ‘.’ 0.1 ‘ ’ 1

Correlation of Fixed Effects:
          (Intr) TrckC2 TrckC3 speed
TrackCat2  0.017
TrackCat3  0.361  0.629
speed     -0.985 -0.145 -0.469
moisture  -0.157  0.728  0.590  0.000
```

Estimated marginal means

```
$emmeans
 TrackCat emmean    SE   df lower.CL upper.CL
 1          77.6  9.73 14.8     56.9     98.4
 2         107.5 10.30 17.9     85.9    129.2
 3         120.2 13.90 32.4     91.9    148.6

Degrees-of-freedom method: kenward-roger
Confidence level used: 0.95

$contrasts
 contrast              estimate   SE   df t.ratio p.value
 TrackCat1 - TrackCat2    -29.9  9.4 25.4  -3.181  0.0104
 TrackCat1 - TrackCat3    -42.6 14.3 26.3  -2.973  0.0166
 TrackCat2 - TrackCat3    -12.7 11.1 26.1  -1.138  0.4998

Degrees-of-freedom method: kenward-roger
P value adjustment: tukey method for comparing a family of 3 estimates
```

Frequency domain

Axial hoof acceleration magnitude

Linear mixed model fit by REML. t-tests use Satterthwaite's method ['lmerModLmerTest']
Formula: axial hoof acceleration magnitude ~ track category + speed + moisture + (1 | Horse)

```
REML criterion at convergence: 326.5

Scaled residuals:
     Min       1Q   Median       3Q      Max
-1.88225 -0.53827 -0.09267  0.51349  2.63008

Random effects:
 Groups   Name        Variance Std.Dev.
 Horse    (Intercept) 158.85   12.60
 Residual              81.35    9.02
Number of obs: 45, groups:  Horse, 11

Fixed effects:
             Estimate Std. Error       df t value Pr(>|t|)
(Intercept) -66.31407   56.67563 34.69788  -1.170   0.2499
TrackCat2     3.54658    4.45458 30.18167   0.796   0.4322
TrackCat3     7.99233    6.53110 31.12443   1.224   0.2302
speed         6.60827    3.40405 34.44596   1.941   0.0604 .
moisture     -0.03561    0.43680 30.22025  -0.082   0.9356
---
Signif. codes:  0 ‘***’ 0.001 ‘**’ 0.01 ‘*’ 0.05 ‘.’ 0.1 ‘ ’ 1

Correlation of Fixed Effects:
          (Intr) TrckC2 TrckC3 speed
TrackCat2 -0.081
TrackCat3  0.275  0.622
speed     -0.982 -0.061 -0.400
moisture  -0.172  0.741  0.633 -0.001
```

Moisture removed
Formula: axial hoof acceleration magnitude ~ track category + speed + (1 | Horse)

```
REML criterion at convergence: 326.7

Scaled residuals:
     Min       1Q   Median       3Q      Max
-1.90755 -0.54069 -0.07519  0.51432  2.65867

Random effects:
 Groups   Name        Variance Std.Dev.
 Horse    (Intercept) 159.16   12.616
 Residual              78.81    8.877
Number of obs: 45, groups:  Horse, 11

Fixed effects:
            Estimate Std. Error     df t value Pr(>|t|)
(Intercept)  -66.769     55.020 35.733  -1.214   0.2329
TrackCat2      3.812      2.946 31.002   1.294   0.2052
TrackCat3      8.318      4.978 32.557   1.671   0.1043
speed          6.588      3.354 35.456   1.964   0.0574 .
---
Signif. codes:  0 ‘***’ 0.001 ‘**’ 0.01 ‘*’ 0.05 ‘.’ 0.1 ‘ ’ 1

Correlation of Fixed Effects:
          (Intr) TrckC2 TrckC3
TrackCat2  0.069
TrackCat3  0.503  0.294
speed     -0.997 -0.090 -0.517
```

Axial Cannon acceleration magnitude

Linear mixed model fit by REML. t-tests use Satterthwaite's method ['lmerModLmerTest']
Formula: axial cannon acceleration magnitude ~ track category + speed + moisture + (1 | Horse)

```
REML criterion at convergence: 284.4

Scaled residuals:
     Min       1Q   Median       3Q      Max
-1.49705 -0.55767 -0.08977  0.43149  2.09951

Random effects:
 Groups   Name        Variance Std.Dev.
 Horse    (Intercept) 83.52    9.139
 Residual             84.48    9.191
Number of obs: 40, groups:  Horse, 11

Fixed effects:
             Estimate Std. Error       df t value Pr(>|t|)
(Intercept) -130.4498    62.4684  33.6485  -2.088   0.0444 *
TrackCat2      5.8780     4.8858  25.8952   1.203   0.2398
TrackCat3      5.1870     7.3428  27.6797   0.706   0.4858
speed          9.9220     3.7828  33.5083   2.623   0.0130 *
moisture       0.3918     0.4570  25.9022   0.857   0.3990
---
Signif. codes:  0 '***' 0.001 '**' 0.01 '*' 0.05 '.' 0.1 ' ' 1

Correlation of Fixed Effects:
          (Intr) TrckC2 TrckC3 speed
TrackCat2  0.016
TrackCat3  0.354  0.628
speed     -0.985 -0.150 -0.467
moisture  -0.152  0.728  0.595 -0.012
```

Moisture removed
Formula: axial cannon acceleration magnitude ~ track category + speed + (1 | Horse)

```
REML criterion at convergence: 285.4

Scaled residuals:
     Min       1Q   Median       3Q      Max
-1.68467 -0.61782 -0.00179  0.49218  2.18878

Random effects:
 Groups   Name        Variance Std.Dev.
 Horse    (Intercept) 84.09    9.17
 Residual             83.54    9.14
Number of obs: 40, groups:  Horse, 11

Fixed effects:
            Estimate Std. Error      df t value Pr(>|t|)
(Intercept) -122.866     61.479  34.670  -1.998   0.0536 .
TrackCat2      2.824      3.330  27.067   0.848   0.4039
TrackCat3      1.416      5.872  29.665   0.241   0.8111
speed          9.995      3.766  34.614   2.654   0.0119 *
---
Signif. codes:  0 '***' 0.001 '**' 0.01 '*' 0.05 '.' 0.1 ' ' 1

Correlation of Fixed Effects:
          (Intr) TrckC2 TrckC3
TrackCat2  0.188
TrackCat3  0.560  0.353
speed     -0.998 -0.207 -0.572
```

Attenuation

Linear mixed model fit by REML. t-tests use Satterthwaite's method ['lmerModLmerTest']
Formula: attenuation ~ track category + speed + moisture + (1 | Horse)

```
REML criterion at convergence: 160

Scaled residuals:
     Min       1Q   Median       3Q      Max
-2.11265 -0.48637  0.02793  0.56851  1.45326

Random effects:
 Groups   Name        Variance Std.Dev.
 Horse    (Intercept) 6.029    2.455
 Residual             1.807    1.344
Number of obs: 40, groups:  Horse, 11

Fixed effects:
            Estimate Std. Error       df t value Pr(>|t|)
(Intercept) -9.72171    9.73514 28.69899  -0.999    0.326
TrackCat2   -0.97279    0.71863 25.23572  -1.354    0.188
TrackCat3   -1.43599    1.09219 25.86455  -1.315    0.200
speed        0.73440    0.58783 28.34650   1.249    0.222
moisture     0.02138    0.06722 25.25034   0.318    0.753

Correlation of Fixed Effects:
          (Intr) TrckC2 TrckC3 speed
TrackCat2  0.017
TrackCat3  0.362  0.629
speed     -0.985 -0.144 -0.469
moisture  -0.158  0.728  0.588  0.003
```

Moisture removed
Formula: attenuation ~ track category + speed + (1 | Horse)

```
REML criterion at convergence: 156.5

Scaled residuals:
     Min       1Q   Median       3Q      Max
-2.20370 -0.53371  0.01855  0.57899  1.52950

Random effects:
 Groups   Name        Variance Std.Dev.
 Horse    (Intercept) 6.025    2.455
 Residual             1.747    1.322
Number of obs: 40, groups:  Horse, 11

Fixed effects:
            Estimate Std. Error      df t value Pr(>|t|)
(Intercept)  -9.3076     9.4627 29.6738  -0.984   0.3333
TrackCat2    -1.1396     0.4848 26.3139  -2.351   0.0265 *
TrackCat3    -1.6424     0.8688 27.2393  -1.890   0.0694 .
speed         0.7383     0.5786 29.3578   1.276   0.2119
---
Signif. codes:  0 ‘***’ 0.001 ‘**’ 0.01 ‘*’ 0.05 ‘.’ 0.1 ‘ ’ 1

Correlation of Fixed Effects:
          (Intr) TrckC2 TrckC3
TrackCat2  0.195
TrackCat3  0.570  0.363
speed     -0.996 -0.213 -0.582
```

Estimated marginal means

```
$emmeans
 TrackCat emmean    SE   df lower.CL upper.CL
 1          2.87 0.803 11.4   1.1150     4.63
 2          1.73 0.833 13.2  -0.0631     3.53
 3          1.23 1.060 26.1  -0.9378     3.40

Degrees-of-freedom method: kenward-roger
Confidence level used: 0.95

$contrasts
 contrast              estimate    SE   df t.ratio p.value
 TrackCat1 - TrackCat2    1.140 0.486 26.4   2.346  0.0667
 TrackCat1 - TrackCat3    1.642 0.875 27.3   1.878  0.1643
 TrackCat2 - TrackCat3    0.503 0.831 26.9   0.605  0.8186

Degrees-of-freedom method: kenward-roger
P value adjustment: tukey method for comparing a family of 3 estimates
```

Resultant hoof acceleration magnitude

Linear mixed model fit by REML. t-tests use Satterthwaite's method ['lmerModLmerTest']
Formula: resultant hoof acceleration magnitude ~ track category + speed + moisture + (1 | Horse)

```
REML criterion at convergence: 399.6

Scaled residuals:
    Min      1Q  Median      3Q     Max
-1.6562 -0.4969 -0.1409  0.4081  2.5292

Random effects:
 Groups   Name        Variance Std.Dev.
 Horse    (Intercept) 1453.3   38.12
 Residual              453.2   21.29
Number of obs: 45, groups:  Horse, 11

Fixed effects:
            Estimate Std. Error       df t value Pr(>|t|)
(Intercept) -498.3257   135.8087  33.5041  -3.669 0.000838 ***
TrackCat2     11.8352    10.5235  30.4008   1.125 0.269544
TrackCat3     15.8548    15.4723  30.9922   1.025 0.313427
speed         35.3710     8.1463  33.0878   4.342 0.000126 ***
moisture       0.6756     1.0320  30.4252   0.655 0.517596
---
Signif. codes:  0 '***' 0.001 '**' 0.01 '*' 0.05 '.' 0.1 ' ' 1

Correlation of Fixed Effects:
          (Intr) TrckC2 TrckC3 speed
TrackCat2 -0.081
TrackCat3  0.277  0.622
speed     -0.981 -0.060 -0.401
moisture  -0.171  0.741  0.632  0.002
```

Moisture removed
Formula: resultant hoof acceleration magnitude ~ track category + speed + (1 | Horse)

```
REML criterion at convergence: 401.9

Scaled residuals:
    Min      1Q  Median      3Q     Max
-1.7263 -0.4411 -0.1402  0.3664  2.7182

Random effects:
 Groups   Name        Variance Std.Dev.
 Horse    (Intercept) 1473.5   38.39
 Residual              443.2   21.05
Number of obs: 45, groups:  Horse, 11

Fixed effects:
            Estimate Std. Error      df t value Pr(>|t|)
(Intercept) -483.782    132.445  34.532  -3.653 0.000852 ***
TrackCat2      6.719      6.988  31.269   0.962 0.343664
TrackCat3      9.385     11.863  32.244   0.791 0.434622
speed         35.406      8.063  34.076   4.391 0.000104 ***
---
Signif. codes:  0 '***' 0.001 '**' 0.01 '*' 0.05 '.' 0.1 ' ' 1

Correlation of Fixed Effects:
          (Intr) TrckC2 TrckC3
TrackCat2  0.070
TrackCat3  0.505  0.295
speed     -0.996 -0.090 -0.519
```

Resultant cannon acceleration magnitude

Linear mixed model fit by REML. t-tests use Satterthwaite's method ['lmerModLmerTest']
Formula: resultant cannon acceleration magnitude ~ track category + speed + moisture + (1 | Horse)

```
REML criterion at convergence: 377.2

Scaled residuals:
    Min      1Q  Median      3Q     Max
-2.1521 -0.4875  0.0649  0.3602  1.5056

Random effects:
 Groups   Name        Variance Std.Dev.
 Horse    (Intercept) 3805.3   61.69
 Residual              823.2   28.69
Number of obs: 40, groups:  Horse, 11

Fixed effects:
            Estimate Std. Error       df t value Pr(>|t|)
(Intercept) -255.292    209.858   27.792  -1.216 0.234027
TrackCat2     49.368     15.349   25.005   3.216 0.003570 **
TrackCat3     93.349     23.366   25.473   3.995 0.000488 ***
speed         16.983     12.657   27.339   1.342 0.190697
moisture       3.165      1.436   25.018   2.204 0.036944 *
---
Signif. codes:  0 '***' 0.001 '**' 0.01 '*' 0.05 '.' 0.1 ' ' 1

Correlation of Fixed Effects:
          (Intr) TrckC2 TrckC3 speed
TrackCat2  0.017
TrackCat3  0.363  0.629
speed     -0.984 -0.143 -0.469
moisture  -0.159  0.728  0.587  0.006
```

Estimated marginal means

```
$emmeans
 TrackCat emmean   SE   df lower.CL upper.CL
 1          84.2 20.5 12.6     39.8      129
 2         133.6 21.2 14.4     88.2      179
 3         177.5 26.2 26.1    123.7      231

Degrees-of-freedom method: kenward-roger
Confidence level used: 0.95

$contrasts
 contrast                estimate   SE   df t.ratio p.value
 TrackCat1 - TrackCat2    -49.4 15.4 25.2  -3.212  0.0097
 TrackCat1 - TrackCat3    -93.3 23.5 25.7  -3.980  0.0014
 TrackCat2 - TrackCat3    -44.0 18.2 25.6  -2.412  0.0586

Degrees-of-freedom method: kenward-roger
P value adjustment: tukey method for comparing a family of 3 estimates
```

Post-hoc assessment of anterior-posterior and medial-lateral hoof accelerations in the time domain

Peak hoof anterior-posterior acceleration

Linear mixed model fit by REML. t-tests use Satterthwaite's method ['lmerModLmerTest']
Formula: AP hoof acceleration ~ track category + speed + moisture + (1 | Horse)

```
REML criterion at convergence: 367.4

Scaled residuals:
    Min      1Q  Median      3Q     Max
-1.5573 -0.5759 -0.0321  0.4760  2.2081

Random effects:
 Groups   Name        Variance Std.Dev.
 Horse    (Intercept) 615.1    24.80
 Residual             205.8    14.35
Number of obs: 45, groups:  Horse, 11

Fixed effects:
            Estimate Std. Error       df t value Pr(>|t|)
(Intercept) -191.4835    91.3502  33.5581  -2.096 0.043687 *
TrackCat2     21.6479     7.0910  30.2680   3.053 0.004691 **
TrackCat3     23.3899    10.4221  30.9053   2.244 0.032120 *
speed         13.1016     5.4808  33.1655   2.390 0.022661 *
moisture       2.7293     0.6954  30.2942   3.925 0.000463 ***
---
Signif. codes:  0 ‘***’ 0.001 ‘**’ 0.01 ‘*’ 0.05 ‘.’ 0.1 ‘ ’ 1

Correlation of Fixed Effects:
          (Intr) TrckC2 TrckC3 speed
TrackCat2 -0.081
TrackCat3  0.277  0.622
speed     -0.981 -0.060 -0.401
moisture  -0.171  0.741  0.632  0.001
```

Estimated marginal means

```
$emmeans
 TrackCat emmean    SE   df lower.CL upper.CL
 1          75.5  8.52 13.7     57.2     93.8
 2          97.2  8.80 15.5     78.5    115.9
 3          98.9 11.10 29.3     76.3    121.5

Degrees-of-freedom method: kenward-roger
Confidence level used: 0.95

$contrasts
 contrast              estimate    SE   df t.ratio p.value
 TrackCat1 - TrackCat2   -21.65  7.10 30.2  -3.050  0.0128
 TrackCat1 - TrackCat3   -23.39 10.50 30.9  -2.236  0.0809
 TrackCat2 - TrackCat3    -1.74  8.22 30.9  -0.212  0.9755

Degrees-of-freedom method: kenward-roger
P value adjustment: tukey method for comparing a family of 3 estimates
```

Peak hoof medial-lateral acceleration

Linear mixed model fit by REML. t-tests use Satterthwaite's method ['lmerModLmerTest']
Formula: ML hoof acceleration ~ track category + speed + moisture + (1 | Horse)

```
REML criterion at convergence: 343.1

Scaled residuals:
     Min       1Q   Median       3Q      Max
-1.76788 -0.61070  0.05884  0.46236  1.81212

Random effects:
 Groups   Name        Variance Std.Dev.
 Horse    (Intercept) 268.3    16.38
 Residual             119.4    10.93
Number of obs: 45, groups:  Horse, 11

Fixed effects:
            Estimate Std. Error      df t value Pr(>|t|)
(Intercept)  48.0876    69.0004 34.2436   0.697   0.4906
TrackCat2    12.6900     5.3990 30.1724   2.350   0.0255 *
TrackCat3    20.8466     7.9229 31.0049   2.631   0.0131 *
speed        -0.7185     4.1430 33.9491  -0.173   0.8633
moisture      0.4586     0.5294 30.2065   0.866   0.3932
---
Signif. codes:  0 ‘***’ 0.001 ‘**’ 0.01 ‘*’ 0.05 ‘.’ 0.1 ‘ ’ 1

Correlation of Fixed Effects:
          (Intr) TrckC2 TrckC3 speed
TrackCat2 -0.081
TrackCat3  0.276  0.622
speed     -0.982 -0.060 -0.401
moisture  -0.172  0.741  0.633  0.000
```

Moisture removed

```
REML criterion at convergence: 344.4

Scaled residuals:
    Min      1Q  Median      3Q     Max
-1.6038 -0.5318  0.0466  0.4205  1.9664

Random effects:
 Groups   Name        Variance Std.Dev.
 Horse    (Intercept) 272.4    16.50
 Residual             118.0    10.86
Number of obs: 45, groups:  Horse, 11

Fixed effects:
            Estimate Std. Error      df t value Pr(>|t|)
(Intercept)  58.4016    67.6286 35.2884   0.864   0.3937
TrackCat2     9.2226     3.6049 30.9932   2.558   0.0156 *
TrackCat3    16.4927     6.1004 32.3766   2.704   0.0108 *
speed        -0.7222     4.1215 34.9678  -0.175   0.8619
---
Signif. codes:  0 ‘***’ 0.001 ‘**’ 0.01 ‘*’ 0.05 ‘.’ 0.1 ‘ ’ 1

Correlation of Fixed Effects:
          (Intr) TrckC2 TrckC3
TrackCat2  0.069
TrackCat3  0.504  0.294
speed     -0.997 -0.090 -0.518
```

```
$emmeans
 TrackCat emmean   SE   df lower.CL upper.CL
 1          46.5 5.51 11.8     34.4     58.5
 2          55.7 5.75 13.8     43.4     68.0
 3          63.0 7.32 28.1     48.0     78.0

Degrees-of-freedom method: kenward-roger
Confidence level used: 0.95

$contrasts
 contrast              estimate   SE   df t.ratio p.value
 TrackCat1 - TrackCat2    -9.22 3.61 31.1  -2.557  0.0403
 TrackCat1 - TrackCat3   -16.49 6.14 32.5  -2.687  0.0295
 TrackCat2 - TrackCat3    -7.27 6.13 32.1  -1.186  0.4702

Degrees-of-freedom method: kenward-roger
P value adjustment: tukey method for comparing a family of 3 estimates
```
